# Circadian Clock Dysfunction Exacerbate Autistic‐Like Behaviour and Wnt/β‐Catenin Signalling Dysregulation in ASD Mice and Treatment of Melatonin

**DOI:** 10.1111/jcmm.70991

**Published:** 2026-01-07

**Authors:** Yuxing Zhang, Yinan Chen, Wu Li, Liya Tang, Guangyu Wang, Jiangshan Li, Xiang Feng

**Affiliations:** ^1^ Hunan University of Chinese Medicine Changsha Hunan Province China; ^2^ Key Laboratory of Hunan Province for Integrated Traditional Chinese and Western Medicine on Prevention and Treatment of Cardio‐Cerebral Diseases Changsha Hunan Province China

**Keywords:** autism spectrum disorders, Bmal1, circadian rhythm, melatonin, Wnt/β‐catenin signalling

## Abstract

Between 50% and 80% of children diagnosed with Autism Spectrum Disorder (ASD) are estimated to experience sleep disturbances, highlighting the importance of exploring the role of the circadian clock in ASD development. Previous studies have identified a potential link between Bmal1 deficiency and ASD in mouse models. In this study, we first characterise the expression patterns of circadian proteins. Subsequent behavioural tests and western blot analyses revealed that mice exposed to valproic acid (VPA) displayed autistic‐like behaviours, along with altered circadian protein expression and disruption in Wnt signalling protein levels. Further studies showed that Bmal1 knockout exacerbates these behavioural changes and further impaired Wnt signalling and downstream protein expression in VPA‐exposed mice. Notably, treatment with the circadian biomarker melatonin reversed Wnt downregulation and improved the behaviour deficit in VPA‐exposed mice. The therapeutic effect of melatonin appears to be mediated by its regulation of the Wnt/β‐catenin signalling pathway, which is linked to Bmal1‐mediated circadian dysfunction. Together, our findings provide experimental evidence supporting the role of circadian dysregulation in ASD pathogenesis, highlight the therapeutic potential of melatonin in VPA‐exposed mice, and suggest that Bmal1 may act as a co‐activator in the Wnt‐β‐catenin signalling pathway.

**Abbreviations**
3 V: Third Ventricle
ASD: Autism spectrum disorder
Bmal1: Brain and Muscle ARNT‐Like 1
Clock: Circadian locomotor output cycles kaput
Co‐IP: Co‐immunoprecipitation
Cre: Cerebellum
Cry: Cryptochrome
DSM‐5: The diagnostic and statistical manual of mental disorders 5
Hpc: Hippocampus
Med obl: Medulla oblongata
MT: Melatonin
Npas2: Neuronal PAS domain protein 2
OC: Optic chiasm
OFT: Open field test
Olf bulb: Olfactory bulb
Per: Period
Pre FC: Prefrontal cortex
SCN: Suprachiasmatic Nucleus
Sup col: Superior colliculus
TTFLs: Transcriptional‐translational feedback loops
VPA: Valproic acid

## 1 Introduction

Autism spectrum disorder (ASD) encompasses a range of early neurodevelopmental conditions characterised by deficits in social communication, repetitive and stereotyped behaviours, and atypical sensory processing [1] (DSM‐5, American Psychiatric Association, 2013). While sleep issues vary among different subtypes and individuals with ASD, a significant majority, ranging from 50% to 80% of children with ASD, experience sleep difficulties [2]. Additionally, individuals with ASD are more likely to have sleep problems compared to typically developing children and those with other neurodevelopmental disorders [3]. Together, these observations suggest a potential link between circadian dysfunction and the pathogenesis of ASD in specific individuals [4].

The circadian clock is widely acknowledged as essential for maintaining overall health, and extensive research has explored the implications of circadian dysfunction during neurodevelopment [5]. In mammals, circadian rhythms are regulated by transcriptional‐translational feedback loops (TTFLs). In these loops, transcription factors such as Clock (Circadian Locomotor Output Cycles Kaput) or Npas2 (Neuronal PAS domain protein 2) form heterodimers with Bmal1(Brain and Muscle ARNT‐Like 1) and translocate to the nucleus, initiating the transcription of Period (Per) and Cryptochrome (Cry) family genes. *De novo* loss‐of‐function variants have been identified in the clock genes *Per1*, *Per2*, *Timeless*, *Bmal1* and *Npas2* among individuals with ASD [6, 7, 8]. Given Bmal1 plays a critical role in regulating mRNA transcription and protein translation, its deficiency has been shown to lead to autistic‐like behavioural changes in mice [9].

Circadian rhythms are modulated by and reciprocally interact with melatonin levels, positioning melatonin as a significant biomarker [10]. Several studies have demonstrated that more than 65% of individuals with ASD exhibit melatonin levels that are less than half of the typical average, interventions targeting melatonin have also garnered increasing research attention [11, 12]. Since 1993, researchers have been studying the effects of circadian biomarker melatonin supplementation in individuals diagnosed with ASD [13, 14]. Recent treatment consensus guidelines recommend the inclusion of melatonin as part of the treatment plan for ASD individuals [15]. melatonin administration in individuals with ASD has been correlated with improvements in sleep parameters, enhanced daytime behaviour and minimal adverse effects [16]. Latest studies suggested that melatonin treatment reduces the oxidative stress and inflammation in the hippocampus of ASD mouse model [17] and benefit to autistic‐like behaviours [18]. However, the mechanism of melatonin has not been elucidated. In particular, the molecular mechanisms of melatonin and related circadian rhythms in the brain need to be further studied.

In this study, we investigated whether the deletion of Bmal1 function exacerbates autism‐like behavioural changes in VPA‐induced mouse model of ASD, aiming to explore therapeutic potential of circadian biomarker melatonin. And for the first time, we demonstrated that Bmal1 act as a co‐activator to facilitate the Wnt‐catenin signalling pathway, target downstream molecules implicated in ASD. Our findings provide new experimental evidence supporting a role for the circadian clock and biomarker melatonin in the development and treatment of ASD.

## 2 Methods and Materials

### 2.1 Animals

All mice used in this study were of the C57BL/6J background. Prior to experimentation, both wide type (WT) and knockout (KO) mice within each group were pretreated in a climatic chamber maintained at an ambient temperature of 24°C ± 2°C and relative humidity of 40%. The light/dark cycle was set to 12/12 h, with zeitgeber time units (ZTs) designated as ZT0 (commencing at 6 a.m.) and ZT12 (starting at 6 p.m.), marking the beginning of the light phase(lights on from 06:00 to 18:00) and the dark phase, respectively, while ensuring free access to food and water. All experimental procedures were approved by the Institutional Animal Care and carried out in accordance with the guidelines for animal experimentation in the Hunan University of Chinese medicine (Ethics No. LLBH‐202304230003).

### 2.2 Generation of Bmal1 KO Mice

Bmal1 conventional knock‐out mice were obtained from Cyagen Biosciences (S‐KO‐01139, Jiangsu, China). Mutant animals were genotyped by PCR using DNA and the following primers: forward primer F1 for WT and KO allele (5′‐ACAAAATGCCATGAAACTCTGGG‐3′); reverse primers R1 for WT allele (5′‐GTAACATGAGCAAGCACAATCAAG‐3′) and R2 for KO allele (5′‐TCAGATTCCTCCTGCAGTTTGATAA‐3′).

### 2.3 Prenatal VPA Model of Autism and Chemicals

The WT and KO mice were mated overnight, respectively, to generate progeny. Following overnight mating, a vaginal plug was inserted into pregnant female mice on embryonic day 0 (E0), and the females were subsequently housed. On E12.5, pregnant mice were administered a single intraperitoneal injection of valproic acid (VPA) at a dose of 500 mg/kg, which was obtained as a white powder from Sigma‐Aldrich (St. Louis, MO, USA). The VPA was dissolved in 0.9% saline to establish an autism spectrum disorder (ASD) mouse model [19, 20].

Melatonin was purchased from Sigma‐Aldrich (Shanghai, China). Melatonin treatment (10 mg/kg body weight) or PBS was injected via intraperitoneal injection once a day over 21 days [21, 22]. Mice were randomly grouped into the melatonin group or PBS group, and the examination was blinded to genotypes. Once behavioural tests were completed, the mice were used in the subsequent experiments as described below.

### 2.4 Open Field Test (OFT)

OFT was used to detect stereotyped behaviour, general locomotor activity and anxiety‐related behaviours in mice. Mouse were allowed to acclimatise to the test room 1 h before conducting the test. Each mouse was placed individually in the centre of a quadrangular apparatus (60 × 60 × 45 cm). The testing apparatus was divided into 16 equal squares and was illuminated by white light. Mouse's behaviour was recorded over 5 min using a webcam. Anxiety‐like behaviour was assessed by measures of time spent in the centre region. The test was performed in a well‐ventilated, darkened and sound/light‐attenuated testing room. The test arena was cleaned with 70% alcohol after each mouse to remove any olfactory cues that may guide the animal's behaviour. All behavioural tests were performed under dim red light (~20 lx at cage level) between Zeitgeber time (ZT) 12~16 (light‐off), when mice were naturally in the active phase. Animals were brought to the behavioural room for habituation 1 h before tests.

### 2.5 Sociability in 3‐Chamber Choice Test

Mice were evaluated for social motivation in a rectangular, 3‐chambered box (60 cm L, 41.5 cm W, 20 cm H) fabricated from clear Plexiglass. Dividing walls had doorways allowing access into each chamber. At the start of the test, the mouse was placed in the middle chamber and allowed to explore for a 10 min habituation period, with the doorways into the two side chambers open. During the habituation phase, no mice showed a preference for either side of the testing arena. After the habituation period, the test mouse was enclosed in the centre compartment of the social test box, and an unfamiliar stranger (a sex‐matched, unfamiliar adult C57BL/6J mouse) was placed in one of the side chambers. The stranger mouse was enclosed in a small Plexiglas cage drilled with holes, which allowed nose contact. An identical empty Plexiglas cage was placed in the opposite side of the chamber. Following placement of the stranger and the empty cage, the doors were re‐opened and the subject was allowed to explore the social test box for a 5‐min session, concluding the sociability phase of the task. The test mouse was returned to the centre chamber and a second stranger mouse was placed in the empty Plexiglass cage. The test mouse was allowed to explore both sides of the chamber for a final 5 min, concluding the social novelty preference phase of the task. An automated image tracking system (Noldus Ethovision) provided measures of time spent within 5 cm proximity to each cage, and entries into each side of the social test box [23].

### 2.6 Morris Water Maze Test

Morris water maze test was utilised to investigate hippocampus‐dependent cognitive spatial learning and memory functions. The test was conducted in a circular pool (1.8 m diameter) filled with water (25°C ± 1°C) to a depth of 40 cm. A platform (10 cm diameter) located in the centre of the northeast quadrant of the pool was submerged 1 cm under the water surface. Both pool and platform were made of polyvinyl plastic, black in colour to prevent intra‐maze cues which may guide escape behaviour. Several constant extra maze visual cues (posters, objects and equipment) were surrounding the maze so that the animal can learn to use the distal cues to navigate a direct path to the submerged platform when starting each trial from a different location. Each mouse received 5 days of spatial training; each day included four trials with different starting points for each trial. The order of the start positions was not repeated throughout the testing days. A trial started by placing a mouse gently into the water tail‐end first, facing the outer edge of the pool. The daily average time spent to reach the platform was calculated, and each trial was terminated when the animal reached the platform and remained above it for 15 s before it would be taken out. On the other hand, if the animal did not reach the platform within 90 s, the animal was gently guided toward the platform and was allowed to sit on it for 15 s. Then, the animals were transferred to a dry holding cage after being dried off with a towel. On the 6th day, a 30‐s probe trial was performed (24 h after the last training session) during which the platform was removed, and mice were placed in a new start position. The time spent within the target quadrant (which formerly contained the platform during training sessions) was presented as a percentage of the total trial length. A camera mounted on the ceiling was placed directly above the pool to record the swim path of each mouse [24].

### 2.7 Cell Culture and Transfection

HEK‐293 T cells and mouse hippocampal HT22 cells were obtained from Proceell (Procell, Wuhan, China); both HEK‐293 T and HT22 were maintained in Dulbecco's Modified Eagle Medium (DMEM) (Procell, Wuhan, China) supplemented with 10% fetal bovine serum (Gibco, Carlsbad, CA, USA) at 37°C in a 5% CO2 humidified incubator. Cells were grown in a monolayer and routinely passaged two or three times per week. Lipofectamine 2000 (Invitrogen, Carlsbad, CA) was used to transfect cells with plasmid DNA. Total RNA and proteins were extracted from 80% confluent cells in culture dishes. To synchronise the rhythmic circadian gene expression, cells were cultured in complete medium containing dexamethasone (100 nmol/L) for 2 h [25, 26]. Then, cells were harvested at the specific time points.

### 2.8 Immunofluorescence Staining

HT22 cells were grown in 24‐well plates to about 30%–45% confluence and were transiently transfected with 1.5 μg of expression plasmid. After 24 h, cells were washed with PBS and fixed with 4% paraformaldehyde (22,329,929, Biosharp, Hefei, China) for 10 min, permeabilized with 0.1% Triton X‐100 for 15 min, and then blocked with 5% BSA in 0.1% PBST for 1 h at room temperature. Cells were incubated overnight at 4°C with primary antibodies, including anti‐β‐catenin (66379–1‐Ig, Proteintech, 1:400) and anti‐Bmal1 (ab230822, Abcam, 1:500). After washing three times with PBS, cells were incubated with corresponding secondary antibodies, including Goat Anti‐Mouse IgG H&L (ab150115, Abcam, 1:500) and Goat Anti‐Rabbit IgG H&L (ab150077, Abcam, 1:500) for 1 h at room temperature. Finally, the slides were incubated with DAPI and an anti‐fluorescence quencher. The slides were analysed using a fluorescence microscope (BX51TRF, Olympus, Japan). Three fields were randomly chosen in every sample, and the fluorescence intensity was analysed using ImageJ software (NIH, Bethesda, MD, USA).

### 2.9 Co‐Immunoprecipitation

HEK‐293 T cells were co‐transfected with the indicated plasmids with lipofectamine 2000 (Invitrogen), and the nuclear and cytoplasmic proteins were extracted. Three kinds of beads were used in this study for Co‐IP assay: Anti‐Myc Magnetic Beads (Beyotime, Shanghai, China); Anti‐Flag Magnetic Beads (Anti‐Flag Magnetic Beads); Protein A/G Magnetic Beads (Elabscience, Wuhan, China). Briefly, the protein extracts were incubated with the equilibrated beads at 4°C overnight with gentle mixing to capture the Flag fusion proteins, Myc fusion proteins, or specific antibody captured proteins. The magnetic beads or agarose beads were collected by placing the tube in the appropriate magnetic separator or by centrifuging. The beads were washed with TBS buffer to remove all the non‐specifically bound proteins. The bound fusion proteins were eluted from the beads with corresponding elution buffer for western blot analysis.

### 2.10 Western Blot

Brain tissues were collected at a specific time point (ZT2–5), and HEK‐293 T and HT22 cells were harvested 6 h after 2 h treatment of dexamethasone (100 nmol/L) [25, 26]. All sampleswere homogenised according to the instructions of the enhanced RIPA cracking liquid (ApplyGEN, Beijing, China), then the protein concentration was measured and balanced among the different samples. After adding 5× loading buffer (CWBIO, Beijing, China), these mixtures were denatured at 100°C for 20 min. 10 μL of protein per lane were separated by 8%, 10%, or 12% sodium dodecyl sulfate‐polyacrylamide gel and transferred to 0.45 mm PVDF membranes (Millipore, Shanghai, China). The membrane was blocked in 5% skim milk for 1 h at room temperature and then incubated with specific antibodies overnight at 4°C. The anti‐Per2 (67513–1‐Ig, Proteintech, 1:5000), anti‐Clock (18094–1‐AP, Proteintech, 1:500), anti‐Nr1d1 (ab174309, Abcam, 1:1500), anti‐Bmal1 (ab230822, Abcam, 1:1000), anti‐RORα (ab207082, Abcam, 1:2000), anti‐Cry1 (13474–1‐AP, Proteintech, 1:2000), anti‐Cry2 (13997–1‐AP, Proteintech, 1:500), anti‐Per1 (13463–1‐AP, Proteintech, 1:500), anti‐Per2 (67513–1‐Ig, Proteintech, 1:5000), anti‐TCF4 (13838–1‐AP, Proteintech, 1:5000), anti‐LaminB1 (12987–1‐AP, Proteintech,1:5000), anti‐β‐catenin (66379–1‐Ig, Proteintech, 1:400), anti‐IgG (30000–0‐AP, Proteintech, 1:2000) were used as the primary antibodies. 1:3000–5000 dilution of the HRP‐linked anti‐IgG (AWS0001b, AWS0002b, Abiowell) was used as the secondary antibody.

### 2.11 Statistical Analyses

Data are presented as mean ± SEM or 95% CI as indicated. Shapiro–Wilk test was employed to evaluate the normality of all datasets. Mean difference comparisons were carried out using Student's *t*‐test (two‐sided; paired where appropriate) between two groups, one‐way ANOVA with post hoc tests, and repeated measures as appropriate. For data that did not conform to a normal distribution, we conducted non‐parametric analyses, Kruskal‐Wallis H test. Determination of acrophase in circadian protein expression was performed by CircWave v3.3 software (developed by Roelof Hut, University of Groningen, Netherlands). CircWave uses a linear harmonic regression fit that describes the data by adding harmonics to the principal wave function. To determine the number of harmonics to add, F‐testing was used for the primary fit and for each added harmonic, with a significance level of 0.001 adopted to reduce the chance of false positives. For statistical comparison of rhythmic characteristics (mesor, phase, amplitude) of protein expression and corticosterone profiles, best‐fit sinusoidal waveforms were generated by regression analyses and shared characteristics tested with equal sum of square F tests (Graphpad Prism 9.3.1).

## 3 Result

### 3.1 Characterisation and Expression Pattern of Circadian Proteins

**FIGURE 1:**
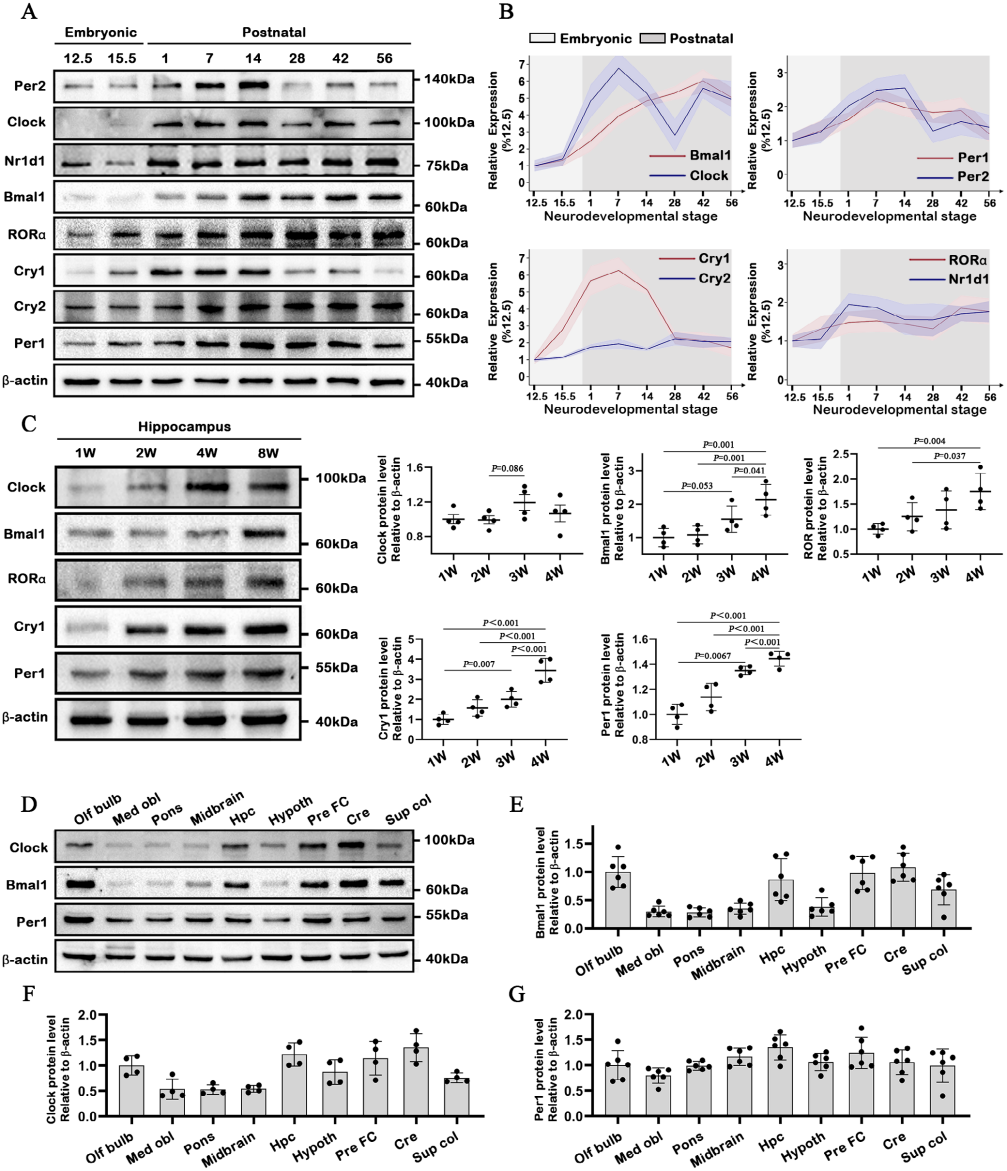
Expression of circadian proteins and characterisation of Clock, Bmal1 and Per1 protein in the developmental C57BL6 mouse brain. (A, B) Developmental expression patterns in mouse brain. C57BL6 mouse brain extracts (30 mg of protein per lane) at the indicated ages (E, embryonic day; P, postnatal day) were analysed by immunoblotting with the indicated antibodies. Actin was monitored as a loading control (*n* = 3). (C) Expression profiles of circadian proteins (Clock, Bmal1, RORα, Cry1 and Per1) in the hippocampus during mouse postnatal development (W, postnatal week) (*n* = 4). (D–G) Tissue distribution of Clock, Bmal1 and Per1 protein in another 6 to 8‐week‐old mouse brain (30 mg of protein per lane). Graph indicates Actin‐normalised Clock, Bmal1 and Per1 protein level. Maximal expression level is adjusted to 100% (*n* = 6). Olf bulb, Olfactory bulb; Med obl, Medulla oblongata; Hpc, Hippocampus; Pre FC, Prefrontal cortex; Cre, Cerebellum; Sup col., Superior colliculus. All data are presented as individual values and mean ± SEM.

In the present work, we first assessed the developmental expression profiles of circadian proteins. In the whole‐brain lysates, Bmal1 and Clock were detected from postnatal day 1 to 56 and peaked between postnatal day 7, 42 and 56. The protein level of Per1, Per2 and Cry1 increased in the first postnatal 2 weeks, while decreasing in the following 3 weeks (Figure 1A,B). The expression of RORα and Nr1d1 protein exhibited a gradual augmentation during the postnatal weeks. In the hippocampus, protein expression of Bmal1, RORα, Cry1 and Per1 exhibited a prominent increase during postnatal development (Figure 1C). In adult C57BL/6 mice, we conducted another experiment to explore the expression pattern of circadian protein in different brain tissues; Clock and Bmal1 protein were abundant in the olfactory bulb, prefrontal cerebral cortex, cerebellum and hippocampus (Figure 1D–G).

### 3.2 Altered Rhythmic Expression of Core Circadian Genes Observed in the VPA‐Induced ASD Mouse Model

**FIGURE 2:**
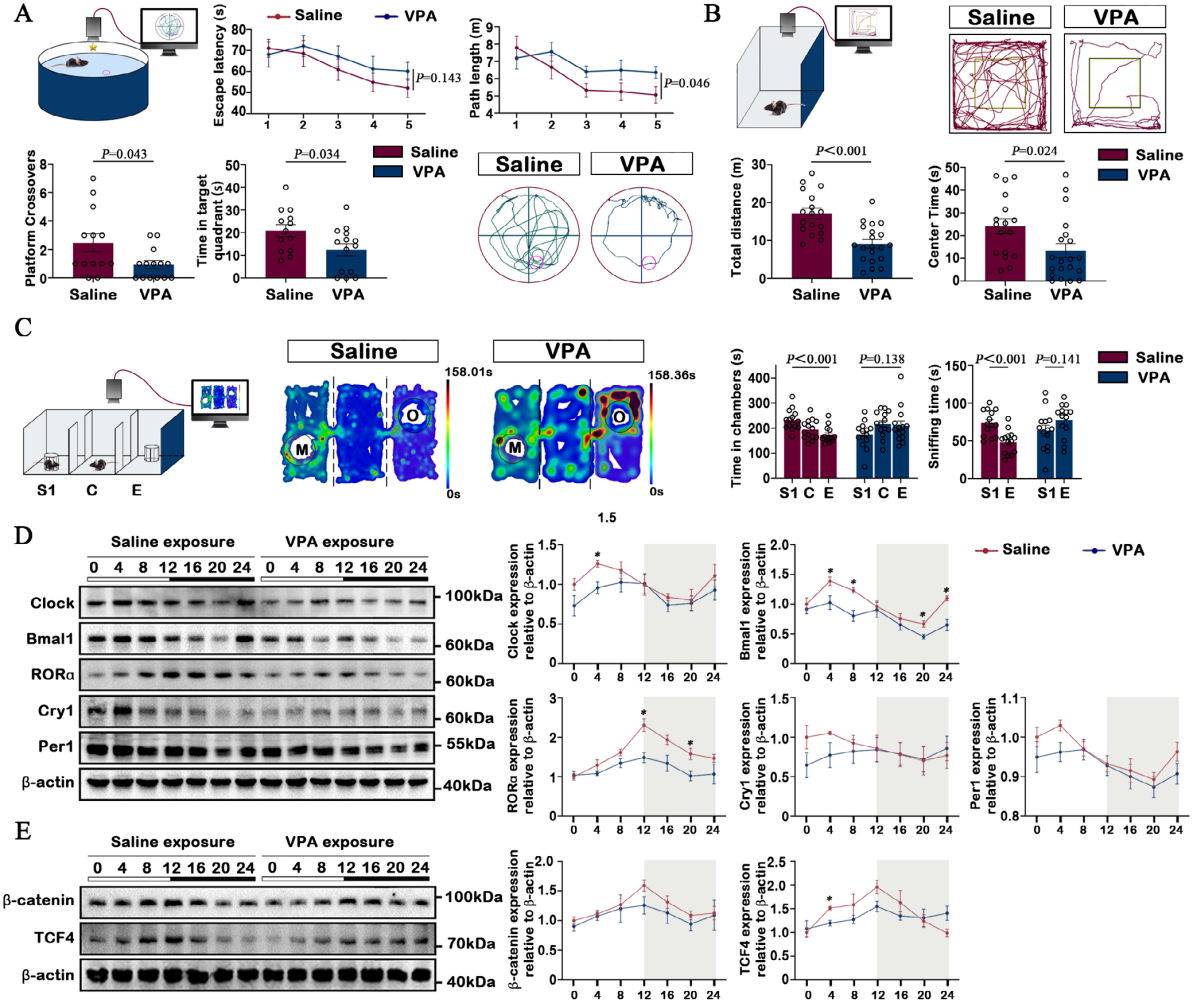
Effects of VPA exposure on the social behaviour of C57BL/6 mice and circadian disruption was observed in VPA exposed mice. (A) Morris water maze test. Top: A schematic diagram of a saline exposed mouse and a VPA exposed mouse in the Morris water maze test, and escape latency, path length (learning phase) line graph. Bottom: Time in target quadrant, platform crossovers were showed in bar graph, and the representative trajectory chart of both two groups (*n* = 13 in Saline exposure group, *n* = 14 in VPA exposure group). (B) Open field test. Top: A schematic diagram and representative trace maps of a saline exposed mouse and a VPA exposed mouse in the open field test. Bottom: Bar graph indicates the total distance and centre time (inside the yellow border) in both two groups (*n* = 13 in Saline exposure group, *n* = 14 in VPA exposure group). (C) Three‐chamber social interaction test. Left: Schematic diagram indicates the setup of the test. Centre: Representative heat maps of two groups. Right: Bar graphs indicating time spent in individual chambers and time spent sniffing wire cages during the three‐chamber test (*n* = 13 in Saline exposure group, *n* = 14 in VPA exposure group). S1: Stranger 1, C: Centre, E: Empty, M: Mice, O: Object. (D, E) Western blot test. Left: Immunoblots indicating the expression of the circadian proteins, including Clock, Bmal1, RORα, Cry1, Per1 and β‐catenin‐TCF4 pathways in the whole mice brain. Right: Quantitation of protein levels of Clock, Bmal1, RORα, Cry1, Per1 and β‐catenin‐TCF4 pathways (*n* = 3 in each time point group). All data are presented as individual values and mean ± SEM. *p < 0.05.

We initially employed a well‐established animal model of ASD, wherein C57BL/6 mice were exposed to Valproic acid (VPA), to investigate the behavioural deficits exhibited in VPA‐exposed mice. VPA‐exposed mice had significantly shorter swim distance and escape latency to find the hidden platform (during training days) than the saline group (Figure 2A). The locomotor activity and anxiety‐like behaviour were assessed using the open field test in each experimental group. It was observed that the VPA group exhibited a significant decrease in both total distances travelled and time spent in the centre compared to the saline group (Figure 2B). The mice exposed to saline spent significantly more time in the chamber with and interacting with a caged unfamiliar mouse (S1), compared to an empty wire cup (E), thus demonstrating robust sociability (Figure 2C). The VPA‐exposed mice, in contrast, displayed impaired social approach behaviour, as evidenced by more similar times spent in the two chambers and sniffing the two wire cages (Figure 2C).

**FIGURE 3:**
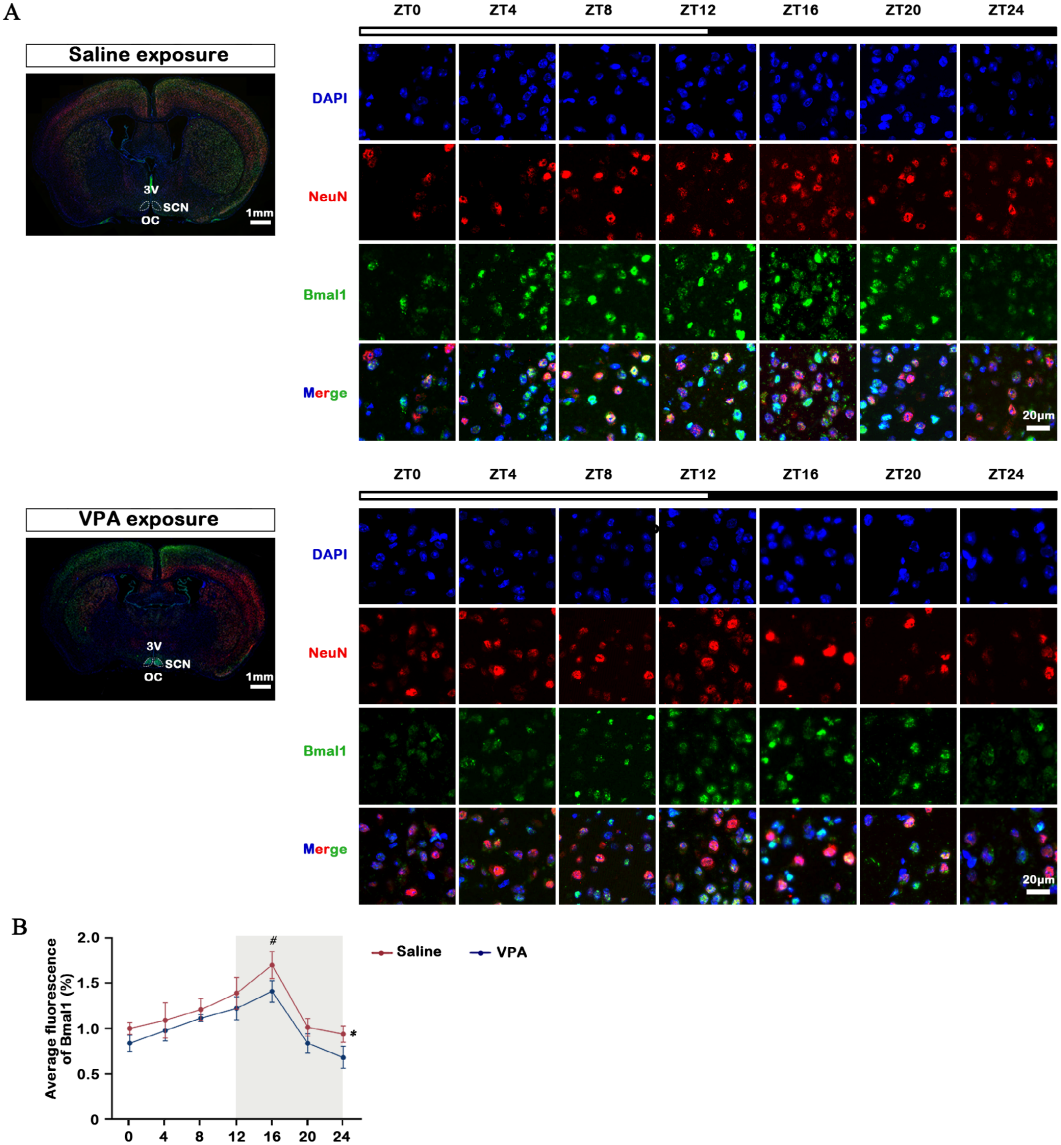
Effects of VPA exposure on the Bmal1 expression in SCN region and serum melatonin of C57BL/6 mice. (A, B) Dual immunofluorescence images and quantitation line chart showed the expression of Bmal1(green) and NeuN (red) in both Saline and VPA exposure group (*n* = 6 each). *Scale bar*, 20 μm. All data are presented as individual values and mean ± SEM. 3 V: Third ventricle; OC: Optic chiasm; SCN: Suprachiasmatic Nucleus. ZT24 values are a duplicate of ZT0 shown for clarity. Genes were identified as rhythmically expressed in unison by CircWave, *p < 0.001 was considered to indicate significant rhythmic expression, ^#^ *p* < 0.05 compared to the VPA group at the same timepoint.

We next examined whether prenatal VPA exposure alters the rhythmic expression patterns and amplitude of core circadian proteins, including Bmal1, Clock, RORα, Cry1 and Per1, across a 24‐h light–dark cycle compared with saline‐treated controls, to determine whether prenatal disruption of the circadian machinery contributes to the molecular and behavioural abnormalities observed in the ASD mouse model. Quantification of Bmal1, Clock, Cry1 and Per1 in the whole brain revealed a peak in expression during the early light phase in both groups, while both the expression level and amplitude were significantly decreased in the VPA group compared to the Saline group. And RORα expression in the whole brain of both groups peaked at the late stage of light exposure, similarly, the expression level and amplitude were significantly decreased in the VPA group compared to Saline group (Figure 2D). The Wnt/β‐catenin signalling pathway has been implicated in neurodevelopmental processes and ASD pathogenesis, and recent evidence suggests potential crosstalk between circadian regulation and Wnt signalling [27, 28]. Therefore, we examined whether prenatal VPA exposure affects the expression of β‐catenin and TCF4, two key transcriptional effectors of the canonical Wnt pathway. β‐catenin expression showed a mild, non‐significant decreasing trend across the circadian cycle, whereas TCF4 expression was significantly reduced at ZT4 and exhibited a dampened rhythmic amplitude compared with saline controls (Figure 2E). These results suggest that prenatal VPA exposure may partially disrupt Wnt/β‐catenin signalling rhythmicity, potentially contributing to the neurodevelopmental abnormalities observed in ASD models. Taken together, these western blot results demonstrate that prenatal VPA exposure disrupts both behaviour and molecular rhythmicity, with Bmal1 showing the most pronounced reduction in rhythmic expression among the core clock genes. Given its central role in circadian regulation and neurodevelopment, Bmal1 was selected as the primary focus of our subsequent mechanistic investigations. Furthermore, our immunofluorescence study focusses on the expression of Bmal1 in the SCN (suprachiasmatic nucleus) region. Dual immunofluorescence analysis indicates that the VPA group exhibited a loss of Bmal1 rhythmic expression in the SCN region (*p* > 0.001), with significant differences in Bmal1 fluorescence intensity at ZT16 (*p* < 0.05) (Figure 3A,B).

In summary, VPA exposure induced robust autism‐like behavioural deficits in mice, including impaired learning, heightened anxiety‐like behaviour and diminished sociability. At the molecular level, VPA disrupted the circadian rhythm by reducing the expression and amplitude of core clock genes (Bmal1, Clock, Cry1, Per1 and RORα) and attenuating β‐catenin/TCF4 signalling, with a marked loss of Bmal1 rhythmicity in the SCN. These findings suggest that circadian disruption and altered Wnt/β‐catenin signalling may underlie the behavioural abnormalities observed in the VPA model of ASD.

### 3.3 Mutation of Bmal1 Aggravates VPA‐Induced Behavioural Deficits and Dysregulate Translational Control Pathways

**FIGURE 4:**
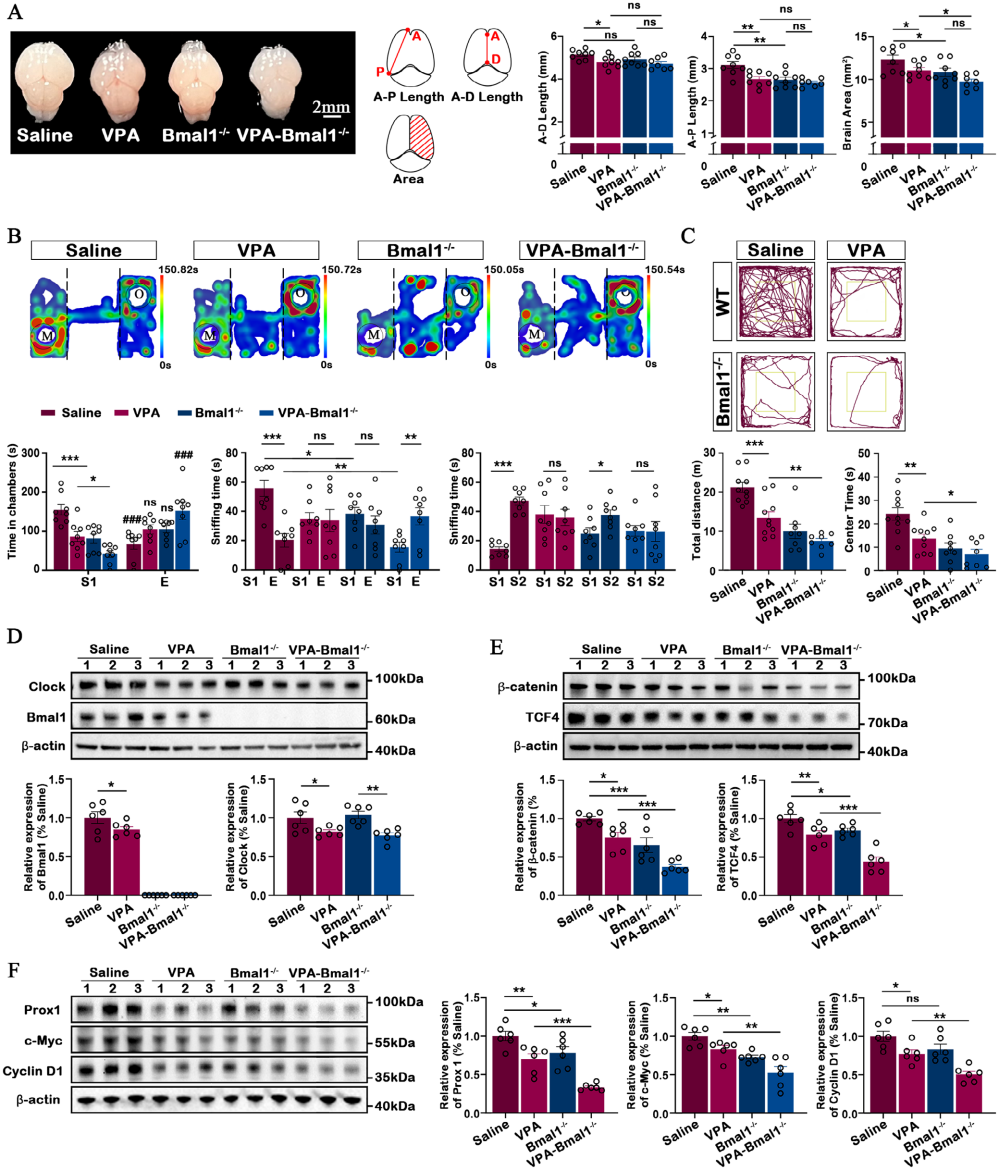
Bmal1 KO aggravated behaviour deficit and dysregulated translational control pathways in the VPA exposure mice. (A) Quantitative comparison of cortical anterior‐dorsal (A‐D) length, anterior‐posterior (A‐P) length and area in Saline group (*n* = 8), VPA group (*n* = 8), Bmal1^−/−^ (*n* = 8) and VPA‐Bmal1^−/−^ mice at P2. (B) Three‐chamber social interaction test. Top: Representative heat maps of two groups. Bottom: Bar graphs indicating time spent in individual chambers and time spent sniffing wire cages during the three‐chamber test (*n* = 8 in each group). S1: Stranger 1, S2: Stranger 2, C: Centre, E: Empty, M: Mice, O: Object. (C) Open field test. Top: Representative trace maps of a saline exposed mouse and a VPA exposed mouse in the open field test. Bottom: Bar graph indicates the total distance and centre time (inside the yellow border) in both two groups (*n* = 8 in each group). (D) Representative immunoblots and quantitation of the Clock and Bmal1 protein (*n* = 6 in each group). β‐Actin was used as a loading control. (E) Representative immunoblots and quantitation of the β‐catenin and TCF4 protein (*n* = 6 in each group). β‐Actin was used as a loading control. (F) Representative immunoblots and quantitation of the Prox1, c‐Myc and Cyclin D1 protein (*n* = 6 in each group). β‐Actin was used as a loading control. All data are presented as individual values and mean ± SEM. *p < 0.05, **p < 0.01, ***p < 0.001. E chamber compared to S1 chamber within the same group ^###^ *p* < 0.001. ns, Non‐significance.

As previously discussed, the circadian system plays a crucial role in the pathogenesis and progression of ASD. Therefore, we aimed to investigate the involvement of Bmal1 in ASD, as its mutation may contribute to autistic‐like behaviours by affecting cerebral development [23]. To determine whether prenatal VPA exposure or Bmal1 deficiency affects gross brain morphology, we assessed overall brain size and cortical dimensions across groups. We observed a mild reduction in cortical length in VPA‐Bmal1^−/−^ mice without reaching statistical significance, while the cortical area showed a significant decrease compared with the VPA group (Figure 4A). These findings suggest that combined VPA exposure and loss of Bmal1 may impair cortical growth, potentially reflecting neurodevelopmental alterations underlying ASD‐like behavioural phenotypes. In the behaviour test, Bmal1^−/−^ mice spent significantly less time in the S1 chamber compared to the E chamber, demonstrating impaired sociability. In addition, after losing the function of gene Bmal1, VPA exposed mice displayed further impaired social approach behaviour, as evidenced by more similar times spent in the two chambers and sniffing. Interestingly, the Bmal1^−/−^ mice spent significantly more time in the chamber with the novel stranger (S2) than in the S1 chamber and more time sniffing the novel stranger cup, indicating a preference for social novelty. However, after losing the function of gene Bmal1, VPA exposed mice spent similar time in the S2 and S1 chamber, demonstrating an impaired preference for social novelty (Figure 4B). Similarly, the locomotor activity and anxiety‐like behaviour were also assessed using the open field. It was observed that the Bmal1^−/−^ group exhibited a significant decrease in both total distances and centre time compared to the saline group (*p* < 0.001). In addition, loss of function of Bmal1 reduces the total distances and centre time in the VPA‐Bmal1^−/−^ group compared to the VPA group (Figure 4C).

The potential mechanism profiling results were further investigated by conducting western blotting to examine the translational control pathways in the forebrain. The expression of Bmal1 was found to be absent in the brains of both Bmal1^−/−^ and VPA‐Bmal1^−/−^ groups, as expected (Figure 4D). The expression of circadian protein Clock in both the VPA and VPA‐Bmal1^−/−^ groups exhibited a significant reduction compared to the saline and Bmal1^−/−^ groups (Figure 4D). The inhibition of the β‐catenin signalling pathway was observed in both VPA and Bmal1^−/−^ groups, as evidenced by the decrease in levels of TCF and β‐catenin proteins. The TCF and β‐catenin protein expression of VPA‐Bmal1^−/−^ group was further significantly decreased than that of VPA group (Figure 4E). In addition, significant reduction of β‐catenin signalling pathway downstream proteins, including Prox1 and c‐Myc, was also detected in both VPA and Bmal1^−/−^ groups; further, VPA‐Bmal1^−/−^ group significantly reduced the expression of Prox1, c‐Myc and Cyclin D1 proteins compared to VPA group (Figure 4F).

### 3.4 Melatonin Treatment Rescues Core Behavioural Deficits and Reverses β‐Actin Inhibition in VPA Exposed Mice by Targeting Circadian

**FIGURE 5:**
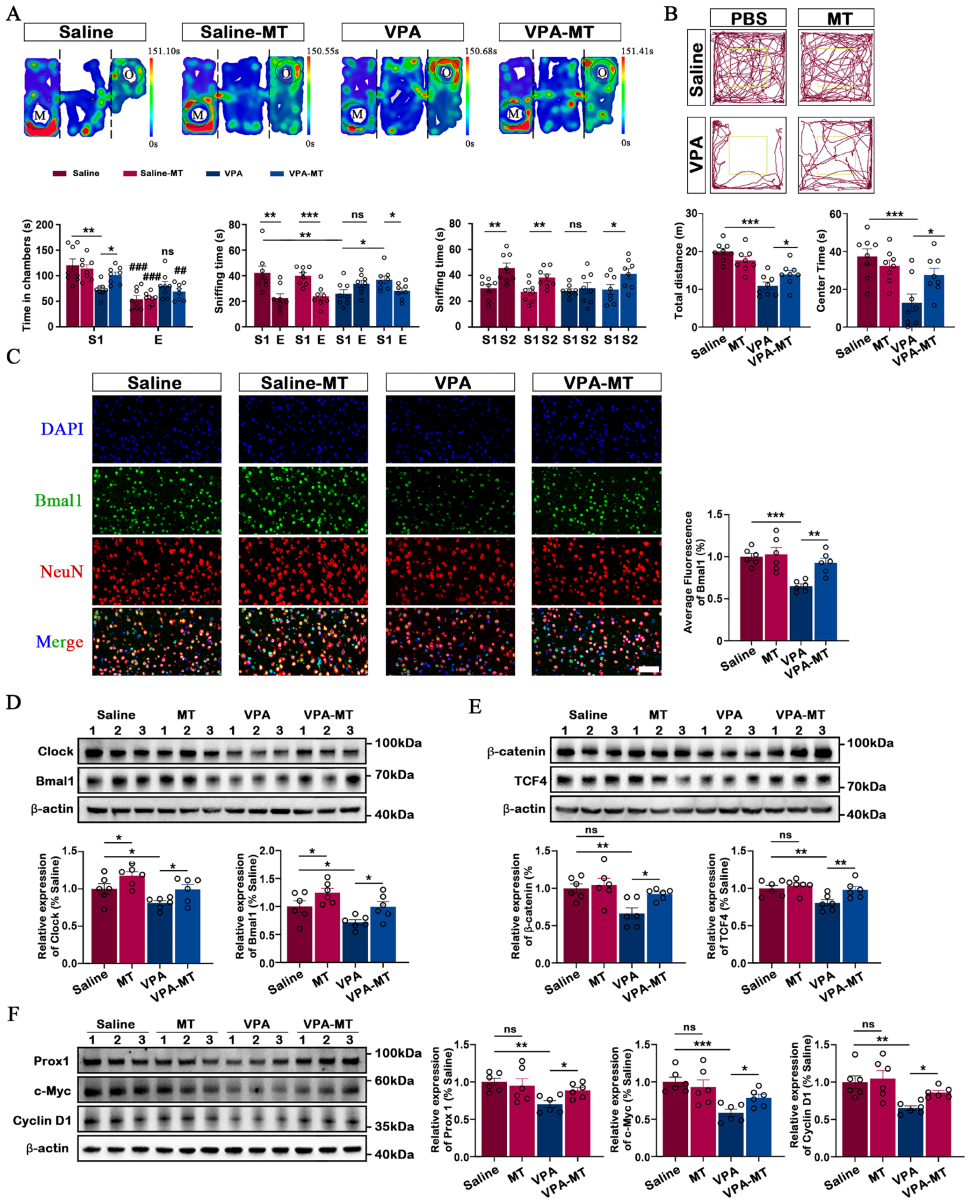
Melatonin ameliorates behavioural deficits and elevate the inhibition of translational control pathways in Bmal1 KO mice. (A) Three‐chamber social interaction test. Top: Representative heat maps of two groups. Bottom: Bar graphs indicating time spent in individual chambers and time spent sniffing wire cages during the three‐chamber test (*n* = 8 in each group). S1: Stranger 1, S2: Stranger 2, C: Centre, E: Empty, M: Mice, O: Object. (B) Open field test. Top: Representative trace maps of a saline exposed mouse and a VPA exposed mouse in the open field test. Bottom: Bar graph indicates the total distance and centre time (inside the yellow border) in both two groups (*n* = 8 in each group). (C) Dual immunofluorescence images and quantitation line chart showed the expression of Bmal1(green) and NeuN(red) in prefrontal cortex (*n* = 6 each). *Scale bar*, 50 μm. (D) Representative immunoblots and quantitation of the Clock and Bmal1 protein (*n* = 6 in each group). β‐Actin was used as a loading control. (E) Representative immunoblots and quantitation of the β‐catenin and TCF4 protein (*n* = 6 in each group). β‐Actin was used as a loading control. (F) Representative immunoblots and quantitation of the Prox1, c‐Myc and Cyclin D1 protein (*n* = 6 in each group). β‐Actin was used as a loading control. All data are presented as individual values and mean ± SEM. *p < 0.05, **p < 0.01, ***p < 0.001. E chamber compared to S1 chamber within the same group ^###^ *p* < 0.001. ns, Non‐significance.

Melatonin treatment reversed the impaired sociability in the VPA group, as evidenced by the significantly increased time spent in the S1 chamber and sniffing the wire cage containing S1 compared to the E chamber (Figure 5A). Additionally, VPA mice treated with melatonin spent significantly more time in the S2 chamber with the novel stranger than in the S1 chamber, indicating a preference for social novelty (Figure 5A). Also, it was observed that the VPA mice treated with melatonin exhibited a significant increase in both total distances and centre time compared to the VPA group (Figure 5B).

Next, we use immunofluorescence to validate the exact molecular changes within the forebrain. Interestingly, double labeling for NeuN and Bmal1 revealed a reduction in Bmal1 protein levels in forebrain neurons in the VPA group, whereas melatonin treatment effectively reversed this inhibition (Figure 5C). The potential mechanism of melatonin was further conducted by western blotting. The expression of circadian protein both Clock and Bmal1 in the VPA groups exhibited a significant reduction compared to the saline and MT groups (Figure 5D). The inhibition of the β‐catenin signalling pathway was observed in the VPA group, while melatonin treatment reverses the reduction of TCF and β‐catenin protein expression when compared to the VPA group (Figure 5E). In addition, significant reduction of β‐catenin signalling pathway downstream proteins, including Prox1 and c‐Myc, were also detected in the VPA group; melatonin also exhibits significantly effects in upregulating the expression of Prox1, c‐Myc and Cyclin D1 proteins compared to the VPA group (Figure 5F).

### 3.5 Bmal1 Acts as a Co‐Activator of Wnt/β‐Catenin Signalling in HT22 Cells

**FIGURE 6:**
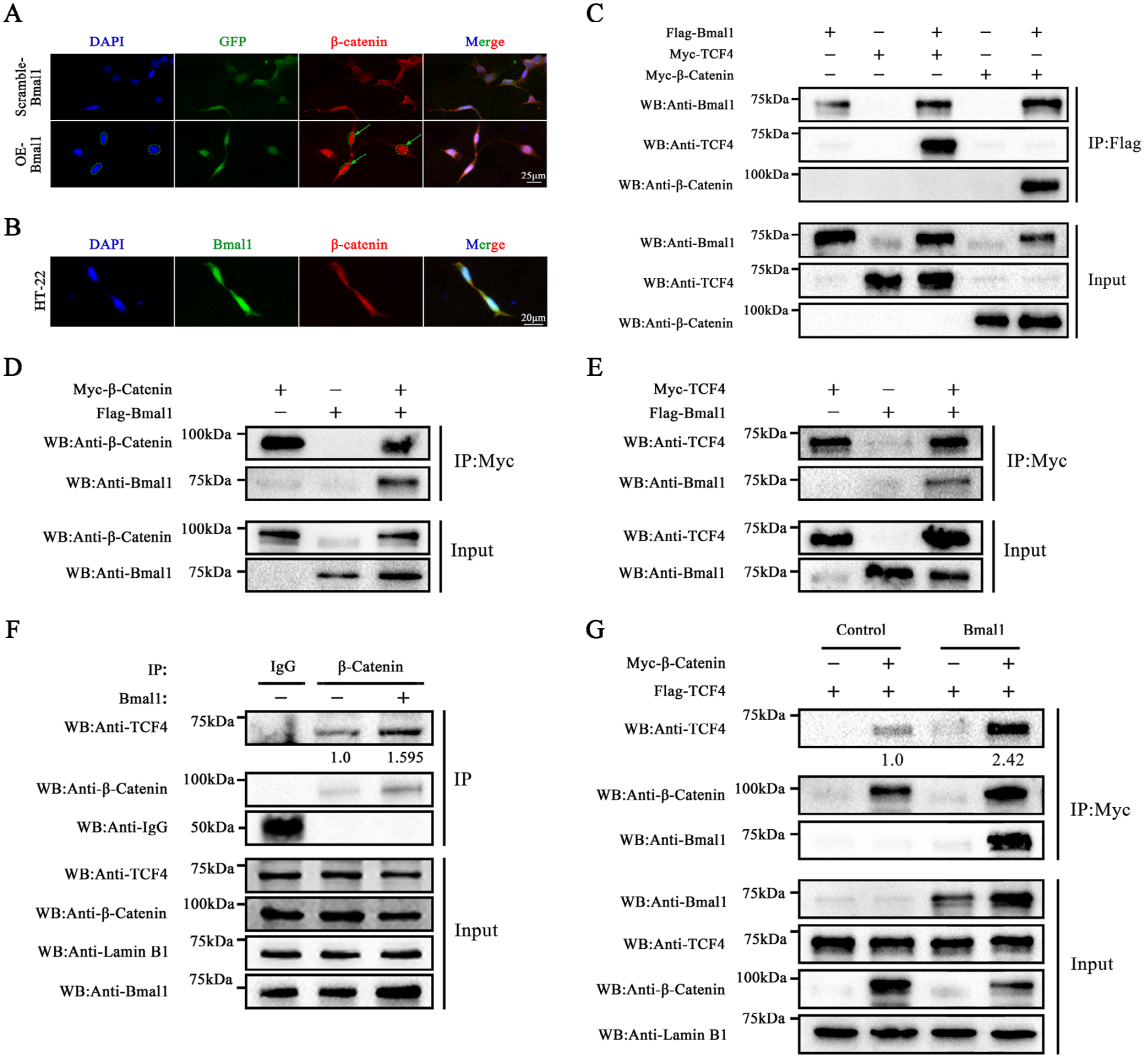
Bmal1 acts as a co‐activator of Wnt/β‐catenin signalling in HT‐22 cells. (A) Immunofluorescence showed overexpression of Bmal1 upregulated the β‐catenin levels and elevates β‐catenin nuclear translocation in HEK‐293 cells. *Scale bar*, 25 μm. (B) Dual immunofluorescence showed Bmal1(green) and β‐catenin(red) Co‐Localise in the Nucleus. Merged channel image shows co‐localization (yellow). *Scale bar*, 20 μm. (C) Co‐immunoprecipitation showed the physical interaction between Bmal1 and TCF4/β‐catenin in HEK‐293 cells co‐expressing Myc‐TCF4, Myc‐β‐catenin and Flag‐Bmal1 by immunoprecipitating Flag beads. (D, E) Co‐immunoprecipitation showed the physical interaction between Bmal1 and TCF4/β‐catenin in HEK‐293 cells co‐expressing Myc‐β‐catenin and Flag‐Bmal1 or Myc‐TCF4 and Flag‐Bmal1 by immunoprecipitating Myc beads. (F) Bmal1 enhances the interaction of endogenous TCF4 and β‐catenin. HT‐22 cells were infected with Bmal1 lentivirus or scramble lentivirus, selected by puromycin. The nuclear fractions were incubated with an anti‐β‐catenin antibody for the IP experiment. IgG was used as a negative control. The immunoprecipitates and the nuclear extracts were subjected to Western blots using the indicated antibodies. Lamin B1 was used to be the loading control of nuclear protein. (G) Bmal1 enhances the interaction of β‐catenin and TCF4. Plasmids of Myc‐β‐catenin, Flag‐TCF4 and Bmal1 were co‐transfected into HEK‐293 cells. The cell lysates were subjected to IP with anti‐Myc beads. The immunoprecipitates and the nuclear proteins were subjected to Western blots using the indicated antibodies. Lamin B1 was used to be the loading control of nuclear protein.

In most cases, the reduced expression of β‐catenin is the cause of inhibiting the canonical pathway. Considering the significant alterations in β‐catenin expression observed in Bmal1^−/−^ mice and VPA mice supplemented with melatonin, we next conducted immunofluorescence staining to elucidate the direct mechanism underlying the interaction between the circadian core gene Bmal1 and β‐catenin signalling. Firstly, β‐catenin protein was mainly located in the nucleus of HT22 cells, overexpression of increased the nuclear expression of β‐catenin protein, indicating that Bmal1may accelerate the nuclear import of β‐catenin (Figure 6A). To determine the co‐localization of Bmal1 and β‐catenin, dual‐immunofluorescence staining of Bmal1 with β‐catenin was performed in HT22 cells. The visualisation of the dual immunofluorescent staining overlap revealed a robust co‐localization of Bmal1 and β‐catenin within the nucleus, as indicated by a prominent yellow signal (Figure 6B).

In the canonical pathway, β‐catenin combined with the LEF/TCF4 complex to activate Wnt signalling, we subsequently examined whether Bmal1 interacted with the nuclear components TCF4 and β‐catenin of the Wnt/β‐catenin signalling pathway separately. Reciprocal immunoprecipitation experiments indicated that Bmal1 interacted with TCF4 and β‐catenin in HEK‐293 T cells (Figure 6C), conversely, both TCF4 and β‐catenin can interact with Bmal1 (Figure 6D,E). Therefore, we hypothesized that Bmal1 may potentiate the assembly of the β‐catenin:TCF4 complex and function as a co‐activator within the Wnt/β‐catenin signalling pathway. Immunoprecipitation experiments indicated that the interaction strength of endogenous β‐catenin and endogenous TCF4 was significantly increased 1.595‐fold in Bmal1‐overexpressed HT22 cells (Figure 6D–F). In HEK‐293 T cells, Co‐IP results showed that the interaction strength of Myc‐β‐catenin with Flag‐TCF4 increased 2.42‐fold in the nucleus when FOXP3 was co‐overexpressed (Figure 6G). These data indicated that Bmal1 acts as a co‐activator of Wnt/β‐catenin signalling to stabilise the interaction strength of the β‐catenin:TCF4 complex in HT22 cells.

## 4 Discussion

In this study, we investigated the involvement of circadian rhythms and melatonin in autistic‐like behaviours and translational control pathways in VPA‐induced ASD mouse models. These findings provide compelling evidence for the role of circadian rhythms in ASD pathology, as well as highlight melatonin as a potential biomarker for improving autistic‐like behaviours. Furthermore, our in vitro results suggest that the underlying mechanism may involve direct interaction between Bmal1 and the β‐catenin:TCF4 complex.

Sleep disturbances are commonly observed in individuals with ASD, who exhibit a higher association with anxiety‐related difficulties in initiating sleep, sleep disorders and disruptions to their circadian rhythm [3, 29]. In addition, a growing amount of research has revealed that circadian rhythm participates in neuronal development, such as conditional knockout Bmal1 in C57BL/6 mice contributing to autistic‐like behaviour and pathological alterations in the cerebellum [23]. The molecular circadian clock in mammals is built upon interlocking transcription‐translation feedback loops (TTFLs), which have a duration of approximately 24 h to complete [30]. A set of interlocked core clock genes and their protein products were involved in circadian biology via TTFLs, including Bmal1, Clock, Cry family and Per family proteins [25]. Numerous articles reported that circadian rhythm is involved in several pathology phenotypes in ASD and other neurodevelopmental diseases, including oxidative stress, synapse plasticity dysfunction and cerebellar dysfunction [31, 32]. Therefore, we first examined the expression patterns of core circadian proteins at different stages of neurodevelopment and across multiple brain regions. As expected, Bmal1 and Clock expression progressively increased with age, suggesting that these molecules may play an essential role in supporting neuronal maturation and the establishment of functional neural circuits. These findings provide the rationale for focusing on Bmal1 as a key molecular link between circadian rhythm dysregulation and ASD‐like phenotypes.

Individuals diagnosed with ASD exhibit difficulties in perceiving the passage of time and possess a limited intuitive sense of circadian rhythms [33]. In addition, abnormal diurnal profiles of cortisol, melatonin and disrupted sleep–wake cycles indicate underlying impairments within the circadian system among individuals with ASD [10]. In this study, we subsequently investigate the diurnal variations in the expression of circadian core proteins and melatonin biomarkers in serum. Based on a previous investigation, we established an ASD model through intraperitoneal VPA injection [34]. As expected, VPA mice exhibited significant ASD‐like behaviours, including altered social interaction, repetitive and stereotyped behaviour. The expression level and amplitude of both the circadian core protein Wnt/β‐catenin signalling and melatonin were diminished in the VPA treated group compared to the Saline group. Additionally, a reduction in neuronal expression levels and amplitudes of the core protein Bmal1 was observed in the SCN region. In previous studies, researchers using proteomics have confirmed that the Wnt pathway is highly enriched and upregulated in ASD mice, while phosphorylated β‐catenin levels exhibit a decreasing trend [27]. This partially aligns with our findings. However, given that the Wnt signalling pathway also exhibits circadian expression differences [35], a more extensive time‐series analysis should be employed in future research to capture these dynamic changes more accurately. The dysregulation of Wnt/β‐catenin dependent transcription in ASD has been extensively studied and found to significantly impact neuronal function and synaptic plasticity mechanisms [36, 37]. Here, we observed dysregulation of the circadian pattern of β‐catenin and TCF4, thereby suggesting a potential association between circadian rhythm and Wnt/β‐catenin signalling pathways in models of ASD.

To further investigate the causal relationship between circadian dysregulation and ASD‐like behaviours, we generated Bmal1‐deficient mice in the VPA‐induced ASD background. Our hypothesis was that if Bmal1 serves a protective role in maintaining neuronal and developmental homeostasis, its loss would exacerbate ASD‐like phenotypes rather than rescue them. Bmal1, as a core circadian transcription factor, is essential for neurodevelopment and cerebellar function, and its deficiency has been linked to behavioural abnormalities and disrupted synaptic signalling [23]. Consistent with this hypothesis, homozygous Bmal1 knockout in VPA‐exposed mice led to aggravated autistic‐like behaviours and anxiety, along with signs of delayed brain maturation compared with VPA mice alone. This finding aligns with previous studies showing that heterozygous Bmal1 deletion (Bmal1^+/−^) increases mTOR activity in the brain, abnormal ultrasonic vocalisations, deficits in social interaction and social novelty, excessive repetitive behaviours, impaired motor coordination [9]. These results together suggest that Bmal1 acts as a crucial regulator of neurodevelopment, and its loss amplifies the behavioural and molecular abnormalities induced by prenatal VPA exposure. Thus, circadian disruption mediated by Bmal1 deficiency may represent a key molecular mechanism underlying ASD pathogenesis.

More than 65% of individuals with ASD exhibit melatonin levels that are less than half of the typical average, interventions targeting melatonin have also garnered increasing research attention [11, 12]. Recently, researchers have been studying the effects of circadian biomarker melatonin supplementation in individuals diagnosed with ASD and ASD models [13, 14]. Several studies have indicated that melatonin plays a protective role in neurodevelopmental disorders by exerting therapeutic effects on early synaptic plasticity and neurotransmitter levels [38]. Also, daily treatment with melatonin (3.0 mg/kg) reduced the excessive grooming of the mutant mice to wild‐type levels and improved activity rhythms in the *Cntnap2* mouse model of ASD [39]. Here, we demonstrated that daily melatonin (10 mg/kg) for 21 days significantly reduces autism‐like behaviours induced by VPA, compared with the Saline group, with decreasing the expression level of β‐catenin, TCF protein and downstream targets. Although the ASD model and the doses of melatonin used differ from those employed in previous studies, the consistent finding across these studies is the potential effect of melatonin in mitigating core autistic‐like behaviour and related symptoms. Furthermore, our study investigated the role of melatonin as a circadian biomarker, demonstrating its significant impact on the brain expression levels of Bmal1, Clock and the corresponding phenotypic changes.

Dephosphorylated β‐catenin undergoes cytoplasmic accumulation, nuclear translocation and subsequent interaction with T‐cell factor/lymphoid enhancer factor (TCF/LEF) to enable Wnt‐respondent gene transcription, containing c‐Myc and Cyclin D1, eventually causing alterations in neuronal development [40, 41, 42]. The subsequent findings revealed that melatonin can enhance the expression of Bmal1 and Clock in the brain of ASD mice. These molecular mechanisms are consistent with previous studies [43, 44]. Therefore, we next explore the downstream signalling pathway responsible for Bmal1 function in ASD and found that the Bmal1‐mediated autistic‐like behaviours could be, at least partly, attributed to the Wnt/β‐catenin signalling, which is critical for the initiation and progression of ASD [27, 45]. Actually, Bmal1 has constantly been regarded as a transcriptional factor functioning in cells. However, the exact role in the regulation of the Wnt/β‐catenin signalling pathway in human cancers is unknown. Our Co‐IP result indicated that Bmal1 could interact with β‐catenin and TCF4 respectively and reciprocally and enhance the function of β‐catenin and TCF4 in the nucleus.

A limitation of the present study is that, although our data demonstrate that melatonin rescues both behavioural and molecular phenotypes in VPA‐exposed mice, we did not directly assess its efficacy in the context of Bmal1 deficiency. Thus, it remains unclear whether the therapeutic benefits of melatonin are strictly dependent on Bmal1 expression, or whether melatonin may act through additional Bmal1‐independent mechanisms. Future studies using VPA‐exposed Bmal1 KO mice will be essential to determine whether melatonin retains its protective effects in the absence of Bmal1 and to clarify the specific contribution of Bmal1 to melatonin‐mediated rescue. Although the VPA‐induced ASD model successfully reproduces core behavioural features such as social deficits, anxiety and repetitive behaviours, it cannot fully capture the genetic and phenotypic heterogeneity of human ASD. Therefore, future studies incorporating complementary approaches—such as genetic or social isolation models—will be essential to better reflect the multifactorial nature of the disorder. In addition, this study did not distinguish between cytoplasmic and nuclear β‐catenin, and therefore cannot directly conclude that Bmal1 promotes its nuclear translocation. Although our data clearly demonstrate the physical interaction between Bmal1 and β‐catenin, the functional consequence of this interaction on transcriptional activation remains to be verified. Future studies employing nuclear fractionation and confocal imaging will be required to clarify whether Bmal1 facilitates β‐catenin nuclear import and downstream transcriptional regulation.

## 5 Conclusion

In summary, we characterised the developmental expression patterns of circadian proteins in the mouse brain. Our comparative analysis revealed significant alteration in both circadian protein expression patterns and Wnt signalling core protein levels in VPA‐exposed mice. Additionally, we observed the overall therapeutic efficacy of melatonin in these mice, suggesting that its potential mechanism relies on Bmal1 acting as a co‐activator in the Wnt‐β‐catenin signalling pathway.

## Author Contributions

**Yuxing Zhang:** data curation (equal), funding acquisition (equal), investigation (equal), writing – original draft (lead), writing – review and editing (lead). **Yinan Chen:** investigation (equal), methodology (equal). **Wu Li:** conceptualization (equal), data curation (equal), supervision (equal). **Liya Tang:** conceptualization (equal), resources (equal), software (equal). **Guangyu Wang:** investigation (equal), methodology (equal), validation (equal), visualization (equal). **Jiangshan Li:** formal analysis (equal), investigation (equal), methodology (equal), project administration (equal), resources (equal), software (equal). **Xiang Feng:** the first corresponding author of this article, formal analysis (equal), funding acquisition (equal), investigation (equal).

## Funding

This work was supported by National Natural Science Foundation of China (82505793), The National Grants Postdoctoral Researchers Program of China (GZC20230784), Natural Science Foundation of Hunan Province (2024JJ6341), Outstanding Youth Project of Hunan Provincial Department of Education (23B0390,24B0358), National Natural Science Foundation Pre‐research Project of Hunan University of Chinese Medicine (2024XJYY07), and Hunan University of Chinese Medicine Undergraduate Research Innovation Foundation (2023BKS085,2025BKS229).

## Ethics Statement

All experimental procedures were approved by the Institutional Animal Care and carried out in accordance with the guidelines for animal experimentation in the Hunan University of Chinese medicine (Ethics No. LLBH‐202304230003).

## Consent

The authors have nothing to report.

## Conflicts of Interest

The authors declare no conflicts of interest.

## Data Availability

The datasets used and/or analyzed during the current study are available from the corresponding author on reasonable request.
